# Molecular and Metabolic Subtypes in Sporadic and Inherited Clear Cell Renal Cell Carcinoma

**DOI:** 10.3390/genes12030388

**Published:** 2021-03-09

**Authors:** Maria F. Czyzyk-Krzeska, Julio A. Landero Figueroa, Shuchi Gulati, John T. Cunningham, Jarek Meller, Behrouz ShamsaeI, Bhargav Vemuri, David R. Plas

**Affiliations:** 1 Department of Cancer Biology, University of Cincinnati, Cincinnati, OH 45267, USA; cunnijn@ucmail.uc.edu (J.T.C.); vemuribv@ucmail.uc.edu (B.V.); plasd@ucmail.uc.edu (D.R.P.); 2 Department of Veterans Affairs, Cincinnati Veteran Affairs Medical Center, Cincinnati, OH 45220, USA; 3 Department of Pharmacology and System Biology, College of Medicine, University of Cincinnati, Cincinnati, OH 45267, USA; landerjo@ucmail.uc.edu (J.A.L.F.); mellerj@ucmail.uc.edu (J.M.); 4 Agilent Metallomics Center of the Americas, Department of Chemistry, University of Cincinnati, Cincinnati, OH 45221, USA; 5 Division of Hematology and Oncology, Department of Medicine, University of Cincinnati, Cincinnati, OH 45267, USA; Gulatisi@ucmail.uc.edu; 6 Department of Biomedical Informatics, University of Cincinnati, Cincinnati, OH 45267, USA; 7 Division of Biomedical Informatics, Cincinnati Children’s Hospital Medical Center, Cincinnati, OH 45229, USA; 8 Division of Biostatistics and Bioinformatics, Department of Environmental and Public Health Sciences, University of Cincinnati, Cincinnati, OH 45267, USA; Shamsabz@ucmail.uc.edu; 9 Department of Electrical Engineering and Computer Science, College of Engineering and Applied Sciences, University of Cincinnati, Cincinnati, OH 45221, USA

**Keywords:** clear cell renal cell carcinoma, VHL, transcriptomics, proteomics, metabolomics, metallomics, precision medicine

## Abstract

The promise of personalized medicine is a therapeutic advance where tumor signatures obtained from different omics platforms, such as genomics, transcriptomics, proteomics, and metabolomics, in addition to environmental factors including metals and metalloids, are used to guide the treatments. Clear cell renal carcinoma (ccRCC), the most common type of kidney cancer, can be sporadic (frequently) or genetic (rare), both characterized by loss of the von Hippel-Lindau (VHL) gene that controls hypoxia inducible factors. Recently, several genomic subtypes were identified with different prognoses. Transcriptomics, proteomics, metabolomics and metallomic data converge on altered metabolism as the principal feature of the disease. However, in view of multiple biochemical alterations and high level of tumor heterogeneity, identification of clearly defined subtypes is necessary for further improvement of treatments. In the future, single-cell combined multi-omics approaches will be the next generation of analyses gaining deeper insights into ccRCC progression and allowing for design of specific signatures, with better prognostic/predictive clinical applications.

## 1. Introduction

Personalized medicine represents a therapeutic advance where specific treatments are established and delivered based on the molecular landscape for an individual patient or a closely defined group of patients. The concept is based on the fact that patients have unique genomic and functional signatures, in addition to the common genomic aberrations. However, identification of clinically effective signature has encountered several limitations. Tumors show complex genetic heterogeneity, both among the same cancers from different patients and within regions of individual tumors. This genetic complexity is further amplified when data from transcriptomics, proteomics, and metabolomics are considered, all affected by various environmental factors. The usefulness of molecular signatures for prediction of treatments in human cancers is hindered by the fact that these signatures are defined by the steady-state levels of measured molecules and do not adequately represent metabolic fluxes and pathway activities. There can also be poor correlation between abundances of molecules identified from different omics. The next generation of system biology approaches will be the integration of data from more than one omics platform. Single-cell omics are likely to improve the efficacy of prognostic and predictive classifiers. 

**Figure 1:**
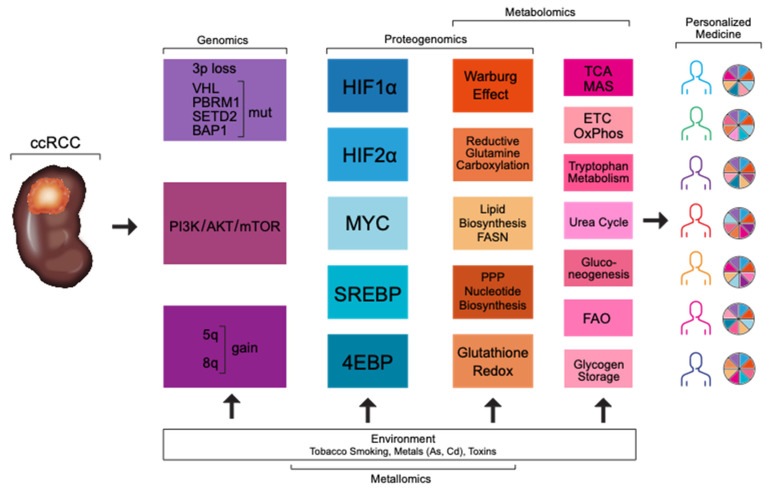
Multiple omics platforms and exposures to the environmental factors are necessary for the identification of ccRCC subtypes with advantages for personalized medicine.

Kidney cancer annually affects about 300,000 patients worldwide and 70,000 in the USA, with 100,000 and 14,000 deaths, respectively. Clear cell renal cell carcinoma (ccRCC) is the most common type of renal cancer. Its canonical genetic feature is inactivation of the gene for the von Hippel–Lindau tumor suppressor (*VHL*) due to the loss of the short arm of chromosome 3p and mutations or hypermethylation of the other allele [1,2,3]. The fundamental function of VHL is targeting hypoxia inducible factors, HIFαs, for degradation, which accumulate in ccRCC, inducing genes essential for cancer progression, including angiogenesis, proliferation, and cancer metabolism. In particular HIF2α is considered an oncogene in ccRCC, while HIF1α likely has a tumor suppressor function and is lost due the frequent deletion of chromosome 14q [4]. However, a more fine-tuned balance between HIF1α and HIF2α has been also proposed. HIF1α is important in tumor initiation and reprogramming of glucose metabolism, while HIF2α regulates biosynthetic pathways such as lipid metabolism, ribosome biogenesis, and transcriptional activity of other factors, such as MYC. Moreover, both HIFs are important for the immune reprogramming of the tumors [5]. Other genes frequently mutated in ccRCC are located on chromosome 3p as well and include: *PBRM1*, a subunit of a SWI/SNF chromatin remodeling complex, mutated in 30–40% [1,2,3,6,7,8,9]; BAP1, an ubiquitin hydrolase, mutated in 10–15% [1,2,3,10,11]; and SETD2 H3K36 methyltransferase, mutated in 10% of ccRCCs [1,2,3,12,13]. Less frequently mutated genes (<6%) include demethylases KDM6A and KDM5C (lysine-specific demethylase 6A and 5C), PTEN, mTOR, PIK3CA, and TP53 [1,2,3]. Importantly, ccRCC is characterized by a unique metabolic phenotype, which both results from and is independent of the genomic alterations. Clearly, histologically defined ccRCC is not a uniform disease, and different molecular and metabolic subtypes are described carrying various therapeutic vulnerabilities with consequences for personalized medicine (Figure 1). 

Surgical resection of the primary renal tumor remains the current standard treatment for localized or locally advanced ccRCC. Approximately 25–30% of ccRCC patients present with metastatic disease at initial diagnosis, while 50% develop metastases after removal of the primary tumors, some at very late time points (20–30 years after surgery) [14,15]. Clinical trials investigating the importance of nephrectomies in metastatic ccRCC are underway, and the role of nephrectomy in metastatic ccRCC is an evolving topic of discussion. In the early 2000s, two clinical trials conducted by the Southwest Oncology Group (SWOG) and European Organization for Research and Treatment of Cancer (EORTC) showed an overall survival benefit when patients underwent cytoreductive nephrectomy prior to receiving systemic therapy with interferon-alpha [16]. More recently, the CARMENA trail showed that systemic therapy alone with a tyrosine kinase inhibitor was not inferior to the combination of a cytoreductive nephrectomy plus systemic therapy in terms of overall survival [17]. The role of cytoreductive nephrectomy when patients are treated with immune checkpoint inhibitors will be answered by the SWOG trial S1931 [18].

Currently, most successful therapies in metastatic RCC are anti-angiogenic drugs to inhibit tumor vascularization [19]. Direct inhibitors of transcription factor HIF2α have been developed, which expands therapeutic targeting to several HIF activities in addition to angiogenesis [20,21]. Two other therapies include inhibitors of the mTOR pathway [22,23], and most recently, therapies to reestablish immune responses to cancer cells [24,25]. In contrast to other solid cancers, there are no therapies targeting cancer cells by drugs with direct cytotoxic activity in ccRCC. Moreover, there are limited data predicting classifications of patients for these treatments in order to apply precision medicine approaches. 

## 2. Genomic Subtypes in Sporadic ccRCC

The recent work from the TRACERx group determined that primary ccRCC tumors are characterized by a high degree of clonal heterogeneity, and identified seven evolutionary molecular subtypes of sporadic ccRCC [2,3]. Analysis of ccRCC evolution reveals initial events that include loss of chromosome 3p, gain of chromosome 5q, and t(3;5) translocation in a mechanism involving chromothripsis, predicated to occur even as early as adolescence [3]. These events occur in a small number of ancestral cancer-initiating cells, which over time acquire additional mutations until full-blown disease is achieved [2]. Three molecular subtypes with rapid progression and multiple metastatic sites include those characterized by: (i) relatively low heterogeneity, high genomic instability, and mutations in multiple clonal drivers (*BAP1*, *PBRM1*, *SETD2*, *PTEN*), besides loss of *VHL*, which result in a clonal sweep during evolution; (ii) mutations in *BAP1* only, in addition to inactivation of *VHL.* This subtype was previously described by the Brugarolas laboratory and correlates with poor survival [10,11]. However, further evaluation revealed that *BAP1* mutations were associated with worse prognosis in female but not male patients [26]. (iii) No mutations in *VHL* and lack of any major driver mutations. This subtype is rare and has a very high proliferation index and often sarcomatoid differentiation. However, other mechanisms that contribute to the inhibition of the VHL pathway are not excluded. Three other subtypes with better prognosis and attenuated progression to solitary metastases are characterized by extensive intratumor heterogeneity and, in addition to *VHL* mutations, mutations in *PBRM1*. These advance to the evolution of clones with mutations in either *SETD2*, PI3K pathway, or somatic copy number alterations. Surprisingly, tumors driven only by mutations in *VHL* have better prognosis and occurrence of single metastases [2]. 

Metastatic tumors were found to have significantly less subclonal alterations compared to the primary tumors, and were enriched for loss of chromosomes 9p21.3 and 14q31.1 [27]. Importantly, the majority of the clonal drivers in metastases are shared by the metastatic and primary tumors, an indication that some of the primary drivers select for the metastasis of competent populations, i.e., subclones with tumor propagating features. A minority of driver clones (5.4%) are specific only for the metastases, an indication that they derived from either a very small number of cells of this subclone in the primary tumor or evolved de novo. In molecular subtypes characterized by low intratumor heterogeneity (multiple drivers, wild type *VHL*, *VHL* and *BAP1* mutations), more ancestral clones were detected in metastases. This implies that the tumor spread might have begun at the early stages of the primary tumor development, with occult micrometastases at the time of surgery. This indicates that metastases will develop independently whether or not the primary tumor is removed, and that the propagating cancer cells are evolutionarily close to the original tumor-initiating cells. In contrast, in the case of tumors with higher clonal heterogeneity, metastatic competence is acquired in some subpopulations later during tumor growth. This indicates that these tumor propagating cells will be close to the most recent clonal population, and that early surgery can prevent metastatic progression. 

## 3. Transcriptomics 

Several gene expression signatures are well characterized in ccRCC, some resulting directly from genomic alterations, and identify vulnerable therapeutic targets. These include angiogenic, metabolic, and proliferative pathways regulated by HIF, MYC, and mTOR. Recently, establishment and deconvolution of immune signatures is sought to help predict responsiveness to immune checkpoint inhibitors. However, little is understood about transcriptomic subtypes that are not related to the genomic subtypes.

The most central pathway relevant for ccRCC are the genes regulated by HIF which are induced by loss of VHL and represent adaptation to a pseudohypoxic environment [19]. These include angiogenic genes, such as VEGFA, which together with its receptor, VEGFR2, became first milestone for successful therapeutic targets that revolutionized treatment of ccRCC patients. VEGF has been targeted by a monoclonal antibody against VEGFA, bevacizumab, while its receptors are targeted with tyrosine kinase inhibitors (TKIs) such as sunitinib, axitinib, and pazopanib. The next generation of TKIs, cabozantinib and lenvatinib, have a broader spectrum and target not only VEGFRs, but also MET, AXL, and FGFR. However, HIF induces other angiogenic factors such as PDGFB, autocrine growth factor TGFα, chemokine SDF, and its receptor CXCR4 [19]. 

The fundamental HIF-related metabolic change is the Warburg effect, i.e., aerobic glycolysis [28]. This is accomplished by induction of glucose transporters, multiple glycolytic genes, and pyruvate dehydrogenase kinase, which phosphorylates and inhibits utilization of pyruvate by mitochondria [29]. This increase in cytosolic glycolysis allows for glucose carbon flux into biosynthetic pathways, such as the pentose phosphate pathway (PPP) leading to nucleotide synthesis, and serine/glycine biosynthesis. In that respect, upregulation of PPP enzymes is a negative prognostic factor in ccRCC [1]. While there is relatively little knowledge of the contribution of mitochondrial oxidative phosphorylation to energy and biosynthesis in ccRCC, HIF induces a switch in the subunits of COX4, stimulating expression of COX4-2 and repressing COX4-1 [30]. This physiological adaptive mechanism supports efficiency of respiration at lower levels of O_2_. However, such replacement can be of consequence for the regulation of oxidative phosphorylation in ccRCC. The high expression of genes encoding enzymes of the TCA cycle, as well as of several genes encoding subunits of respiratory complexes is a positive prognostic factor in ccRCC [1]. Another metabolic event, indirectly reprogrammed by HIFs, is induction of reductive glutamine carboxylation, reversing the flow of carbon from glutamine into citrate and lipid biosynthesis [31,32,33]. Lipid synthesis is an important aspect of ccRCC metabolism, as ccRCC accumulate lipids which together with glycogen contribute to the clear cell phenotype. In the early stages of ccRCC, HIF1 induces expression of lipid transporters resulting in accumulation of extracellular lipids [34]. This is accompanied by HIF1- and HIF2-dependent inhibition of the rate limiting mitochondrial fatty acid transporter, carnitine palmitoyltransferase 1A (CPTAA), reducing activity of fatty acid oxidation and deposition of lipids into the lipid droplets [35]. More advanced tumors show high expression of fatty acid synthase (FASN), an indication of de novo lipid synthesis. FASN is a negative prognostic factor in ccRCC [1]. Lipid synthesis is required for extensive membrane formation by actively dividing cells. Consistently, downregulation of AMPK and increased ACC activity which contribute to lipid synthesis are correlated with worse survival [1]. 

In addition to the Warburg effect, ccRCC shows unique profiles of metabolic genes. A large number of metabolic genes are differentially expressed between ccRCCs and normal kidney tissues, with the majority of genes downregulated [36]. These include genes involved in nucleotide metabolism, glycerolipid metabolism, TCA cycle enzymes, oxidative phosphorylation, inositol metabolism, genes in the metabolism of branched chain amino acids (cysteine, methionine, glycine, serine, and threonine), while genes involved in aspartate, glutamate, and glutamine metabolism, which are upregulated in other cancers, are not changed in ccRCC. Mechanistically, loss of some metabolic genes is related to their localization on chromosome 3p frequently lost in ccRCC, and to indirect effects of the loss of *VHL* [36]. Similarly, repression of gluconeogenesis and urea cycle enzymes was determined in ccRCC [37,38]. The functional consequence of this reprogramming leads to a decrease in redundancy in the metabolic network and utilization of a relatively small set of genes by ccRCC to maintain pathway activities. 

Another important pathway mutated in almost 30% of ccRCCs is the PI3K/AKT/mTOR pathway [2,39]. Mutually exclusive genetic alterations are spread across 20 genes in the pathway, resulting in its high activity in ccRCC. mTORC1 stimulates several programs supporting cell growth and proliferation, including protein translation and turnover, and metabolic anabolic activities. mTORC2 phosphorylates AKT and promotes cell survival and proliferation by phosphorylation and inhibition of FoxO1/3a transcription factors [40]. In particular, mTORC1 activates nucleotide and lipid synthesis, as well glycolysis by its translational effects on HIF1α. De novo lipid synthesis is promoted by the activation of the SREBP transcription factor that controls both lipid and cholesterol synthesis through multiple mTORC1-activated mechanisms, including S6K1, lipin 1, and the CREB regulated transcription coactivator, CRTC2. Similarly, nucleotide de novo synthesis is activated by multiple mTORC1 downstream mechanisms. These include S6K1-dependent phosphorylation of carbamoyl-phosphate synthetase, CAD, an essential enzyme in the initial steps of de novo pyrimidine biosynthesis, ATF4-induced expression of mitochondrial methylenetetrahydrofolate dehydrogenase, MTHFD2, which provides one carbon units for purine synthesis. Moreover, mTORC1 stimulates purinosome assembly, and through downstream transcription factors SREBP and Myc induces expression of enzymes from the pentose phosphate pathway, and transcriptional and translational levels. This positive effect on nucleotide synthesis partners with the strong mTORC1 effects on protein translation through phosphorylation of 4EBP and ribosomal protein S6 by S6K1 and S6K2, and promotes overall ribosomal biogenesis. In that respect, rapalogs such as everolimus and temsirolimus are used for treatment of advanced and metastatic RCC, however with limited efficacy [39,41]. Temsirolimus was the first mTOR inhibitor to be approved in 2007 for use in patients with advanced RCC based on a phase III trial [42]. The drug prolonged overall survival when compared with interferon-alpha, but was associated with significant adverse events. While previously temsirolimus was recommended as the first-line option in patients with poor risk disease, it is now being replaced by other better tolerated drugs. The mTOR inhibitor everolimus, when combined with VEGF-TKI lenvatinib, was FDA approved based on results from a Phase II trial, where the combination improved progression free survival [43]. This combination is part of the guidelines for use in the second line and beyond setting in patients with advanced ccRCC. 

The proto-oncogene c-MYC (MYC) is a basic helix-loop-helix leucine zipper transcription factor conserved in metazoans that is hyperactivated in a wide variety of solid tumors and hematological malignancies. In ccRCC, there are two mechanisms that have been hypothesized to promote MYC hyperactivation. First, there is a proportion of patients that have tumors harboring focal amplifications in chromosome 8q where the MYC gene resides, possibly driving increases in MYC expression [1,44,45]. Of clinical significance, copy number gain of 8q has been found to be associated with a high risk of lymph node and distant metastases. This genetic event serves as an independent prognostic factor [46]. The second mode of MYC hyperactivation occurs as a consequence of loss of *VHL*. HIF-2α activation potentiates MYC’s transcriptional activity [47,48]. HIFs can also directly induce MYC gene expression in the case of a single nucleotide polymorphism in the MYC promoter associated with risk for renal cancer [49]. Conversely, however, HIF-1α appears to be a negative regulator of MYC in ccRCC [50]. Notably, this interplay between MYC and HIF factors in ccRCC tumors may have important ramifications for treatment selection, as tumors from patients that have high levels of MYC and HIF-2α protein are more likely to be refractory to the anti-angiogenic drug sunitinib. This finding is consistent with the previous observation that tumors with transcriptomic signatures reflecting high MYC expression are less responsive to sunitinib therapy [51,52]. The functional significance of the MYC/HIF cooperation has also been more firmly established in vivo through generation of a genetically engineered mouse model with kidney-specific *Vhl* and *Cdkn2a* deletion and doxycycline-regulatable enforced *Myc* overexpression, which produces tumors that recapitulate several features of human ccRCC [53]. Finally, mTORC1 and MYC converge with HIF1α further contributing to the activation of the Warburg effect. mTORC1 supports HIF1α translation [54,55,56], while MYC partners with HIF to cooperatively induce expression of glycolytic genes [57,58].

However, despite this in-depth understanding of the signaling and metabolic gene expression profiles in ccRCC, identification of prognostic subtypes based on differential gene expression profiles is limited. The unsupervised clustering of a large cohort of ccRCCs by The Cancer Genome Atlas (TCGA) Research Network presented in a seminal Nature paper in 2013 divided ccRCC into four prototype clusters of tumors with differential survival [1]. The m1 subtype with the best survival was characterized by a high number of PBRM1 mutations. The m3 subtype with the worst survival showed mutation in PTEN and deletion of CDKN2A. Subtype 4, also with poor prognosis, showed mutation in BAP1, DNA repair genes, and mTOR mutations. There was partial overlap of these clusters of tumors with the stratification accomplished by using ccA/ccB classification approach [59]. Another study used a set of glucose-related genes to stratify ccRCC into two subtypes with significantly different survivals. Upregulation of three genes, PFKP, FBP1, and RGN and downregulation of GYG2, LGALSN1, and KAT2A are positive prognostic signatures [60]. 

In addition to the prognostic applications of the transcriptomic signatures, there is an urgent need to identify signatures predicting responses to treatments, based on the characterization of the primary tumors. Four robust molecular subtypes were identified with significantly different responses of the metastatic disease to sunitinib [51]. The subtypes ccrcc1 and ccrcc4 were enriched for non-responders to sunitinib treatment, showed amplification in the upstream region of MYC, hypomethylation of MYC gene, and overexpression of MYC targets. The ccrcc4 showed also sarcomatoid differentiation and inflammatory and immune suppressive microenvironment, and the highest level of BAP1 mutations, while ccrcc1 had the highest number of SETD2 mutations. In contrast, ccrcc2 and ccrcc3 were enriched for the responders to sunitinib, and ccrcc3 showed transcriptomics similar to the normal kidney tissues and lack of hypoxic markers [51].

Recent analysis of a large cohort of tumors from the IMmotion151 study identified seven different molecular subtypes characterized by different combinations of mutations in several tumor suppressors, including CDKN2A/2B, and transcriptional signatures defining different biological functions [61]. Two of these subtypes were enriched for angiogenic genes; one was also associated with fatty acid oxidation genes; and three subtypes were described by enrichment of cell cycle genes, two of which were also associated with fatty acid synthesis, and one additionally enriched with T-effector, interferon gene expression modules, and PD-L1 staining [61]. Importantly, the subtypes associated with proliferative genes and fatty acid synthesis were responsive to combined therapy using VEGF and immune checkpoint inhibition (atezolizumab + bevacizumab) [61].

These data further support the need for in-depth stratification based on biological functions. In view of the fact that gene expression is regulated not only by genomic alterations, but is also influenced by environmental factors, it is likely that identification of valid prognostic transcriptomics signatures will require rigorous stratification of patients, taking into consideration gender, ethnicity, risk factors, environmental exposures, co-morbidities, and medications. In particular, there is strong evidence that immune profiles of ccRCCs correlate with survival and responses to treatments [62] and immune check point blockade is promising in ccRCC [24,25]. There is growing evidence of specific metabolic requirements and metabolic exchanges between immune and cancer cells that likely contribute to the responsiveness to treatments [63]. However, systematic evaluation of immune heterogeneity and of the correlations between infiltrating immune cells and cancer cells molecular and metabolic profiles is still ongoing, and in view of its complexity will likely require analyses of multiple sample from individual tumors and single cell approaches. 

## 4. Proteomics 

Transcriptomics data are good predictors of gene expression; however, final protein steady-state levels and function are additionally regulated at posttranscriptional stages, including mRNA transport and stability, protein translation, degradation, and posttranslational modifications. Current data regarding ccRCC proteomics are more limited compared to transcriptomics. TCGA includes limited protein analysis that was performed using a selected set of genes by a reverse-phase protein array (RPPA). A recent study performed by the Clinical Proteomic Tumor Analysis Consortium (CPTAC) analyzed a cohort of 103 ccRCCs and 83 normal kidney tissues by mass spectrometry proteome profiling that included posttranslational modifications, parallel transcriptomics, genomics, and DNA methylation [64]. Overall, there was a good global correlation between steady-state levels of proteins and respective mRNAs. Significant positive Spearman correlations were determined in 74% of mRNA-protein pairs in ccRCCs and 52% of pairs in normal kidney tissues. As expected, good mRNA-protein correlation was found in the case of glycolytic genes. However, there are important and relevant sets of genes where this co-regulation is changed in ccRCC. The most striking example of such uncoupling are genes encoding subunits of the mitochondrial electron transport chain complexes and ribosomal and splicing related genes. This is in addition to an overall decrease in the levels of proteins encoding genes involved in oxidative phosphorylation, fatty acid oxidation, and the TCA cycle [64,65,66]. In the case of the TCA cycle, the highest decrease was observed for the malate dehydrogenase 2 (MDH2) and aconitase 2 [65]. However, the differences in mRNA-protein correlation in tumors are additionally affected by tumor grade, loss of chromosome 14, or BAP1 mutations [64]. This points to the possibility that a more comprehensive analysis of ccRCCs may reveal patterns of fine-tuned coordinated coexpression of mRNAs and protein products with functional consequences for tumor progression.

## 5. Metabolomics 

Metabolomic analyses of ccRCC are primarily based on the LC- and GC- mass spectrometry measurement of steady-state levels of metabolites in tumor and normal kidney tissue specimens, with less data comparing tumors stratified by different factors including determinants of progression. The conclusions drawn based on the metabolites’ abundances are clearly limited as the metabolite levels are affected by the activity of the input and output pathways. Studies using ^13^C labeled glucose to trace carbon flux are difficult to perform in patients and have their own limitations in terms of tracer penetrance and full equilibration of the isotope labeled glucose conversions. Recently, biochemical pathway activity was determined by intercorrelations among metabolite abundances [67,68,69].

Clearly, the most fundamental signature of the Warburg effect consistent with genetic and transcriptomic alterations is an increased level of carbohydrates, in particularly glycolytic metabolites [37,67,70,71,72,73]. Some report an increase in metabolites in upper glycolysis, including glucose, glucose 6-phosphate, and fructose 6-phosphate, but decreased metabolites from lower glycolysis [70,71,72,73]. This, together with the increased metabolites of the pentose phosphate pathway measured in several studies, is an indication for the partitioning of the flux of glucose carbon into the nucleotide biosynthetic pathways [67,70,71,72,73]. 

The connection between the glycolytic flux of carbon into the TCA cycle is limited due to the HIF-induced expression of pyruvate dehydrogenase kinase, which phosphorylates and inhibits conversion of pyruvate into acetyl-CoA [29]. Similarly, ccRCC show decreased expression of pyruvate carboxylase that converts pyruvate to oxaloacetate and is an alternative entry of pyruvate into the TCA cycle [70]. However, an increase in the levels of citrate, aconitate, and succinate were seen, while a decrease in malate and fumarate were also demonstrated [72,73]. This implies anaplerotic and cataplerotic reprogramming. A relevant example of anaplerotic reprogramming is the entry of glutamine to the TCA cycle through glutamate and ketoglutarate, which can then convert to succinyl-CoA or be in the process of reductive carboxylation to generate citrate in the mechanism depending on HIF activity [31,32,33]. Glutaminase inhibitor, telaglenastat (CB-839) has been shown to be effective in RCC based on early phase clinical trials. The initial phase I trials tested telaglenastat in combination with everolimus and cabozantinib in patients with heavily pre-treated clear cell and papillary RCC [74,75]. Randomized controlled Phase II trials (ENTRATA and CANTATA) are currently testing telaglenastat with mTOR inhibitors everolimus and cabozantinib, respectively [76]. Results from one of these trials (ENTRATA) were recently presented. The study drugs prolonged progression free survival with a tolerable safety profile. Overall survival data from this trial is awaited. On the other hand, a recent press release from the CANTATA team (https://www.globenewswire.com/fr/news-release/2021/01/04/2152519/0/en/Calithera-Biosciences-Reports-CANTATA-Study-of-Telaglenastat-in-Renal-Cell-Carcinoma-Did-Not-Achieve-Primary-Endpoint.html (accessed on 8 March 2021).) revealed that the combination of telaglenastat and cabozantinib failed to meet its primary endpoint. 

Gene expression and coexpression of mRNAs and proteins for genes involved in oxidative phosphorylation and mitochondrial electron transport chain are overall decreased in ccRCCs as compared to normal kidney tissues [64]. However, there are multiple data supporting reprogramming rather than shutdown of respiratory chain activity. In that respect, HIF1α induces expression of subunit of respiratory complex I, NDUFA4L2, and subunit of complex IV, COX4-2, while supporting degradation of its paralog, COX4-1, in order to optimize oxygen consumption and generation of reactive oxygen species [30,72,77]. Activation of NDUFA4L2 inhibits complex I activity and oxygen consumption [72,77] and high expression of NDUFA4L2 mRNA and protein correlates with tumor stage and worse survival in ccRCC [72,78]. However, integrated analysis of gene expression and metabolomics demonstrated that ccRCCs from lifetime tobacco smokers have diminished activity of glycolysis, increased oxidative phosphorylation mainly through the malate entry via malate aspartate shuttle, MAS, and activity of malate dehydrogenase 2 [67]. MAS, using two antiporters, SLC25A11 and SLC25A13, imports malate and glutamate into the mitochondria, while exporting ketoglutarate and aspartate [67]. Importantly, the metabolic subtype defined by tobacco smoking appears to have better prognosis when the TCGA database is analyzed [67]. Thus, further analysis of oxidative phosphorylation and mitochondrial function in ccRCC may allow for identification of subtypes relevant for precision medicine stratification of patients in respect to the available therapies targeting the mitochondrial respiratory chain [79,80]. 

The in vivo labeling of ccRCC with ^13^C glucose before nephrectomies and tracing the flux of ^13^C confirmed strong labeling of glycolytic intermediates, including pyruvate and lactate, as well as conversion of pyruvate to alanine [81]. Moreover, there was limited entry of glucose-derived carbon into the TCA cycle. The enrichment for labeled acetyl-CoA was less than 5% in ccRCC as compared to 11% in glioblastomas [81]. However, it is not clear if such differences can be related to the duration of hypoxia/ischemia during surgeries removing tumors from different organs.

An important aspect of ccRCC metabolism is biosynthesis of glutathione and maintenance of the ratio of reduced to oxidized glutathione (GSH/GSSG) for management of oxidative stress. Interestingly, more advanced tumors contain higher levels of glutathione-related metabolites, and have higher GSH/GSSG ratios. Importantly, glutathione biosynthesis requires glutamate. Reprogramming of glutamine metabolism via glutamate into the glutathione is proposed in higher grade tumors in order to diminish oxidative stress and promote tumor progression [70,73]. Glutathione synthesis is also supported by activation of the pentose phosphate pathway (PPP) in tumors with worse prognosis, where PPP-derived NADPH is used for reduction of oxidized glutathione. 

Another hallmark of ccRCC is reprogrammed lipid metabolism. The clear cell histological feature of ccRCC results from accumulation of lipids, primarily cholesterol esters, in the cytoplasm. These are subsequently removed during histological processing, creating empty “clear” space. This is accompanied by several transcriptomic events, some regulated by HIFs. Integrated lipidomic and transcriptomic analysis revealed increased accumulation of polyunsaturated fatty acids (PUFA) and overall fatty acid desaturation and elongation [82]. Interestingly, obesity is a risk factor in ccRCC, but paradoxically, patients with ccRCC who are obese have better prognosis [83,84,85]. While mechanistic insights explaining this paradox are not fully explained, the role of fatty acid synthase (FASN) in tumor cells [84] and role of peritumor adipose tissue and inflammation [85] were reported. Tumor cells rely more on de novo lipid synthesis rather than utilization of exogenous lipids, and high levels of FASN are associated with poor prognosis. Elevated lipid synthesis is also supported by reductive carboxylation of glutamine and PPP-generated NADPH required for fatty acid synthesis. Moreover, there is inhibition of fatty acid oxidation which contributes to the lower activity of the TCA cycle. Interestingly, however, during tumor progression there is a reversal in this pathway and higher stage tumors show decreased lipid content and a decrease in citrate which serves as the precursor metabolite for lipid synthesis [70,73]. This can be related to augmented glutathione synthesis and a shift in utilization of NADPH and glutamine away from lipid synthesis. These data imply lipid remodeling through activity of FASN as part of ccRCC progression. In that respect inhibitors of FASN are beginning to be evaluated in cancer [86].

ccRCCs also display a metabolic immunosuppressing signature composed of tryptophan metabolites, kynurenine and quinolinate, with an increased ratio of kynurenine to tryptophan in tumors [67,70,87], urine [88] and serum [89]. Increased kynurenine levels correspond with worse survival and are induced in a subset of patients treated with immune checkpoint inhibitors, such as nivolumab [89]. Importantly however, inhibitors of two enzymes involved in tryptophan metabolism, indoleamine 2,3-dioxygenase (IDO) and tryptophan 2,3-dioxygnease (TDO) are currently in clinical trials with potential applications for treatment of ccRCC. Tryptophan metabolism targeting drugs were initially seen to have positive results in early phase I and II clinical trials. The selective IDO1 inhibitor, epacadostat, when combined with pembrolizumab led encouraging results (partial response or stable disease) in 7 of 11 (63.6%) previously treated patients with kidney cancer [90]. The subsequent phase III trial, ECHO-302 in patients with RCC compared pembrolizumab plus epacadostat versus pazopanib or sunitinib was unfortunately terminated early due to failure of the same drug in patients with melanoma [91,92]. However, due to the strong preclinical rationale, other agents (potent IDO inhibitors such as linrodostat, and the long-acting IDO1 inhibitor KHK2455) are being developed for testing in patients with advanced kidney cancer [93].

Identification of metabolic subtypes with biochemical vulnerabilities targetable by small molecules is important for the advancement of precision medicine in ccRCC. However, a potential limitation in the identification of true metabolic subtypes is the lack of correlation between metabolomic, proteomic, and transcriptomic profiles as indicated in some studies. A lack of correlation was seen between transcriptomic data for metabolic genes in the TCGA cohort and metabolomic data in the MSK cohort, as well as in subsets of tumors analyzed simultaneously for both in the MSK cohort [73]. There was also lack of correlation between mRNAs and protein of the oxidative phosphorylation genes [64]. However, another study using transcriptomics, metabolomics, and metallomics demonstrated robust correlation between the results from each omics, supporting activation of oxidative phosphorylation in tumors from smokers [67]. The important difference between these two studies is that in the later study metabolomic activities and pathways were performed using correlational analysis of metabolites’ abundances rather than a simple evaluation of the steady-state levels.

## 6. Metallomics 

Metals play a major role in multiple cellular processes as constituents of enzymes and transcription factors. Metallomics is based on a comprehensive chemical analysis aimed to quantitatively determine different forms of metals and metalloids within biological systems. It relies on coupling the molecular separation capabilities of liquid or gas chromatography (LC, GC) with inductively coupled plasma mass spectrometry (ICP-MS) to simultaneously separate and measure multiple elements within biological tissues in a wide range of concentrations. The analysis can include essential trace metals and metalloids that contribute to different physiological processes and toxic metals that interfere with them. ICP-MS metallomics in cancer research has been used for the evaluation of the pharmacokinetics and toxicity of approved and proposed metallo-drugs (platinum, ruthenium, and nanoparticle-based drugs) [94,95,96]. Noninvasive techniques like positron emission tomography (PET) scans have been also used to identify global or localized flux of metals like Cu and Fe, relevant in altered cancer metabolism [97,98].

The use of total metal analysis to classify tumors and to differentiate them from healthy tissues has been proposed for certain cancer types. However, metallomics requires fresh frozen tissues, which are not always collected. Therefore, several studies focused metallomics analyses on blood and urine specimens. Recent work showed that ccRCCs have a very different metallomic landscape as compared to normal kidney tissues, with an overall decrease in almost all metal content, which is also distinctly affected by tobacco smoking [67]. The exception is iron with higher tumor content. However, that can be related to tumor vascularization and high numbers of red blood cells, thus not representing iron content in tumor cells. Importantly, distribution of copper to the cytochrome oxidase complex IV determined by SEC-ICP-MS was consistent with other omic determinations of the activation of oxidative phosphorylation in tumors from long-term smokers [67]. Major differences in cadmium and arsenic, including arsenic speciation, were determined with the implication of better understanding of tumor etiology and identification of targetable therapeutic vulnerabilities [67]. Thus, metallomic analyses can expand the data obtained from more traditional omics. 

Iron and copper are essential transition metals that undergo one-electron redox processes used in hydrolases, oxidases, hydroxylases, and other metabolically important enzymes. The most notable cellular process that relies on copper and iron is cellular respiration. Iron-sulfur clusters are found in several components of the electron transfer chain. Copper is essential for the transfer of an electron to molecular oxygen by cytochrome oxidase complex IV in the mitochondrial respiratory chain. Notably, iron is a cofactor of ketoglutarate-dependent dioxygenases. These include hydroxylases of proline and lysine, but also other amino acids such as asparagine, aspartate, and histidine. Proline hydroxylation by iron and ketoglutarate-dependent proline hydroxylases is an essential event leading to recognition by VHL and the proteasomal degradation of the HIFαs [99]. Other iron utilizing ketoglutarate-dependent dioxygenases include the enzyme-regulating epigenome, such as TET dioxygenases that remove the methyl group from pyrimidine bases in DNA [100], Jumonji domain containing histone lysine demethylases [101], and AlkB-type dioxygenases that are DNA repair enzymes performing oxidative dealkylation [102]. Copper has a more limited number of contingent enzymes when compared with iron. An important and relevant example is lysyl oxidase (LOX), an enzyme crucial for collagen maturation, crosslinking collagen and elastin fibers, which is considered to be an unfavorable prognostic marker in ccRCC (The Human Protein Atlas) and, mitochondrial superoxide dismutase SOD2. Studies show the role of iron, iron-binding proteins, and ferroptosis in ccRCC progression [103,104,105,106]. Similarly, the role of the copper transporters in ccRCC advancement was established [107,108]. However, while bioavailability of these metals can be targeted by systemic chelators against cancerous cells in vitro, the in vivo applications suffer from lack of specificity and have largely failed to demonstrate clinical efficacy. The use of trace elements and expression profiles of genes regulating metal metabolism can be used as biomarkers of cancer progression. They have been investigated in some cancers [109] but the speciation of iron or copper in this context has yet to be explored. 

A broader metallomics approach based on chemical speciation of relevant metals offers several advantages over measurement of total metal content. The different pools of metalloproteins in their holoforms can be quantitatively described in cellular fractions, with insight into their involvement in cellular processes. Metallomics offers the possibility to correlate fractions, rather than the total levels of metals, to unveil and confirm observations made from metabolomics and transcriptomics data. In addition to traditional studies of cellular remodeling of metabolic phenotypes, information about mis-metalation with toxic metals like arsenic, mercury, cadmium, and silver that can significantly alter cancer metabolism, can only be noted and studied with the assistance of chromatographic methods coupled to atomic spectroscopy. The multi-elemental capabilities of this approach were key to elucidate a smoking-dependent metabolic phonotype in ccRCC, involving cadmium, a major toxicant present in tobacco products [67]. The integration of metallomics to proteomics, transcriptomics, and metabolomics represents a major technical challenge, as native conditions are necessary in the analysis workflow. Yet when accomplished, it offers a new dimension to the data interpretation.

## 7. ccRCC in VHL Disease 

VHL disease is a rare genetic multiple organ cancer syndrome resulting from the autosomal dominant germline mutations in one allele of *VHL*, followed by the loss of the second allele during the lifetime, with full penetrance by the age of 65. It was originally defined by three types of familial cancers: hemangioblastoma that develops in the central nervous system and retina, ccRCC, and pheochromocytoma. Recently additional lesions were associated with VHL disease: pancreatic cysts and neuroendocrine tumors, endolymphatic sac tumors of the inner ear, epididymal cystadenoma, and broad ligament cystadenoma associated with the male and female reproductive tract. VHL disease is subdivided into subtypes. Type 1 disease, with mutations leading to loss of VHL protein, has high risk of ccRCC but without pheochromocytoma. Type 2 has missense mutations and frequent occurrence of pheochromocytomas, and is further subclassified into three subtypes, of which type 2B also has risk for ccRCC [110]. ccRCC in VHL disease is characterized by multiple bilateral recurring tumors and cysts that require active surveillance and surgical removal when the tumors reach a size of 3 cm, as at this point the metastatic potential is drastically augmented [111]. 

Analysis of multiple tumors derived from individual patients determined that tumors are clonally independent and show different genomic alterations in addition to the loss of *VHL* function [3,112,113]. Similar to sporadic tumors, ccRCCs in VHL disease show chromosomal aberrations involving frequent chromothripsis-like rearrangement between chromosome 3p and 5q resulting in the loss of 3p and gain of 5q [3]. However, these effects vary in individual tumors. Each tumor has different additional somatic mutations, an indication that after common loss of VHL there are different evolutionary trajectories during tumor progression. This is reminiscent of intratumor heterogeneity of sporadic ccRCC, except that different clones form individual separate tumors, yet with similar mutation patterns to the subclones in sporadic ccRCC. Thus, pharmacological treatment is limited by the different landscapes of individual tumors. VEGF-targeted therapies have been studied in VHL disease-associated RCC. Sunitinib was studied in this population in a phase II trial, where partial responses were seen in 6 of 18 patients [114]. Pazopinib was also tested in a phase II trial of 31 patients with VHL disease-associated RCC and a lesional response rate of ~50% was seen [115]. More recently, the HIF-2α inhibitor, MK-6482, was granted accelerated approval by the FDA to treat patients with VHL disease-associated RCC (with nonmetastatic RCC tumors less than 3 centimeters in size, not in need for immediate surgery). The approval came after the results of a phase II trial were presented by Jonasch et al. with an overall response rate of 27.9% in VHL associated RCC and responses seen in other VHL lesions as well [116]. 

## 8. Moving Forwards

Identification of ccRCC molecular subtypes represents first step in the precision medicine approaches for treatment of this cancer. However, it is essential to provide more in-depth identification of subtypes, where the effects of genomic alterations and environmental factors converge on the final functional outputs such as proteomics and protein post-translational modifications. Clearly integrating analyses across multiple omics domains is essential for the improvement of subtype classifications. The integration and state-of-the-art multi -omics analysis approaches, such as multi-omics extensions of NMF or related factorization approaches, or Canonical Correlation Analysis Comparison, would lead to further noise reduction through joint analyses with orthogonal/complementary signals/domains. Subsequently, one might expect to see improved subtype identification through integrated analyses, which is the subject of future studies. 

However, the major limitation in the treatment of ccRCC, even if molecular, metabolic, and functional subtypes are well defined, is intratumor heterogeneity that occurs at the level of genome, transcriptome, and functional outputs that are additionally affected by environmental and microenvironmental factors. Use of next-generation sequencing technologies demonstrates that at least eight biopsies are required to gain full understanding of genomic heterogeneity [2]. In addition, tumor progression results in the selection of specific clonal subpopulations, of which some evolve early and some late. Metachronous metastatic tumors are usually not sequenced. Thus, finding therapeutic approaches targeting multiple cell types and longitudinal tumor progression is a challenge.

Further progress in the treatment of advanced ccRCC will require application of single cell RNA-seq technology in order to identify multiple clonally heterogenous subpopulations of cancer cells with transcriptomic signatures predictive of selective treatment, leading to therapies simultaneously targeting different subclones. This approach will also identify other cell types that contribute to the tumor microenvironment. The studies will need to analyze several regions from the tumors in order to gain insight into spatial functional heterogeneity. In the future, single-cell combined multi-omics approaches will be the next generation of analyses, gaining deeper insights into ccRCC progression.
